# Active Colitis Attenuates Ventricular Excitation–Contraction Coupling by T-Tubular Remodeling

**DOI:** 10.3390/biom16040503

**Published:** 2026-03-27

**Authors:** Edward J. Ouille V, Carlos H. Pereira, Ygor Marinho, Giedrius Kanaporis, Kathrin Banach

**Affiliations:** 1 Department of Internal Medicine/Cardiology, Rush University Medical Center, 1750 W. Harrison St., Chicago, IL 60612, USA; eddieouille5@gmail.com (E.J.O.V.); h85.carlos@gmail.com (C.H.P.); ygor_marinhoferreiradossantos@rush.edu (Y.M.); 2 Department of Physiology & Biophysics, Rush University Medical Center, 1750 W. Harrison St., Chicago, IL 60612, USA; giedrius_kanaporis@rush.edu

**Keywords:** inflammatory bowel disease, ventricular contraction, t-tubular remodeling, junctophilin-2, calpain, angiotensin II

## Abstract

In patients, extraintestinal manifestations of inflammatory bowel disease (IBD) are attenuated ventricular contractile function and arrhythmia. To determine the mechanism of IBD-induced changes in ventricular function, we used a mouse model of dextran sodium sulfate (3.5% weight/volume; 7 days)-induced colitis. Changes in cardiac function were quantified in isolated ventricular myocytes (VM) by cell shortening, imaging of [Ca^2+^]_i_, reactive oxygen species (ROS), and t-tubular density. During colitis, VMs exhibited attenuated cell-shortening and altered Ca^2+^-handling properties. A prolonged Ca^2+^ transient (CaT) rise time correlated with an increased coefficient of variation in the subcellular Ca^2+^ release and an attenuated t-tubular density. T-tubular loss was accompanied by increased ROS production, calpain-2 (CAPN2) expression, junctophilin-2 (JPH-2) cleavage, and autophagy. Inhibition of Angiotensin-converting enzyme during colitis (Perindopril: 3 mg/kg/day) prevented the increase in CAPN2, ROS production, autophagy, and t-tubular remodeling. It failed, however, to restore full length JPH-2. We conclude that, during IBD, the angiotensin II (AngII)-induced loss of t-tubular integrity and altered cellular Ca^2+^ handling can be prevented by suppression of the AngII-dependent increase in CAPN2 and autophagy and thus suppression of AngII signaling might benefit IBD patients with cardiac manifestations of the disease.

## 1. Introduction

Globally, over 4.9 million people suffer from inflammatory bowel disease (IBD) with an expected rise in prevalence to ~1.0% between 2010 and 2030. IBD is a chronic inflammatory disease of the gastrointestinal (GI) tract that includes both ulcerative colitis (UC) and Crohn’s disease (CD). IBD not only causes GI damage, but elicits extraintestinal manifestations, especially during the active phase of the disease [1]. Changes in ventricular function include an increase in global radial strain, diffuse fibrosis, and inflammation [2], as well as an attenuated ventricular contractile strength (left ventricular ejection fraction). Ventricular tachycardia, fibrillation, cardiac conduction disorders (QT prolongation), and sudden cardiac death have also been reported [3,4,5,6]. While the risk for an attenuated cardiac function has been shown, the mechanisms by which colitis results in ventricular remodeling and an increased risk for arrhythmia and attenuated contractile function remain unknown.

Cardiac contractile function depends on the action potential (AP)-induced calcium (Ca^2+^) transient (CaT) amplitude and kinetics as well as activation of the contractile filaments [2]. The CaT is initiated by the activation of the L-type Ca^2+^ channel (LCC) that allows for an influx of Ca^2+^ into the cell. The rise in sub-sarcolemmal [Ca^2+^]_i_ activates the cardiac type 2 ryanodine receptor (RyR2) and initiates the release of Ca^2+^ from the sarcoplasmic reticulum (SR) in a process termed Ca^2+^-induced Ca^2+^-release (CICR). The CaT rise time and its amplitude critically depend on the close spatial and functional interaction of LCC and RyR2 in a so-called Ca^2+^-release unit [2].

The homogeneous rise of [Ca^2+^]_i_ throughout the cytosol of the ventricular myocyte (VM) is ensured by an extensive t-tubular system [3,4]. These invaginations of the plasma membrane run transversely and longitudinally throughout the cell and allow the formation of Ca^2+^ release units not only in the periphery of the cell, but also throughout the interior of the myocyte [4]. A key regulator of the t-tubular structure and LCC–RyR2 interaction is the protein Junctophilin (JPH) which belongs to a family of structural proteins that connect the plasma membrane to intracellular organelles [5]. JPH-2, the isoform predominantly expressed in the heart, interacts with LCC and RyR, thereby stabilizing the Ca^2+^ release unit. Cardiac specific deletion of JPH-2 leads to cardiac dilation and a loss of the t-tubular structure [6,7]. A disruption of the t-tubular network results in an increased subcellular heterogeneity of the AP-induced CICR, a prolonged CaT rise time, reduced CaT amplitude, and an attenuated contractile strength [4,8]. It remains to be determined if colitis-induced loss of contractile strength is caused by subcellular remodeling.

We recently demonstrated for the first time in a mouse model of dextran sodium sulfate (DSS)-induced colitis, an increased propensity for ventricular alternans on the cellular and organ level during the active phase of the disease [9]. This beat-to-beat alternation of the ventricular AP and/or CaT increases the risk for spontaneous arrhythmic trigger events as well as reentry of excitation [10]. These changes were the consequence of a colitis-induced increase in Angiotensin II (AngII) signaling [9]. In the heart, AngII through the AngII receptor type 1 (AT1R) increases the production of reactive oxygen species (ROS). ROS activates cellular autophagy [11,12] which plays a central role in t-tubular maintenance. In this study, using the animal model DSS-induced active colitis, we tested the hypothesis that an attenuated ventricular contractile function during active colitis is induced by an AngII dependent destabilization and degradation of the t-tubular structure.

## 2. Materials and Methods

### 2.1. Animals and Animal Models

In male C57BL/6 mice (3–5 months; The Jackson Laboratory, Bar Harbor, ME, USA) active colitis was induced by supplementing drinking water for 7 days with 3.5% weight/volume (*w*/*v*) Dextran Sodium Sulfate (DSS; MW: 36–50 kDa, MP Biomedicals, Irvine, CA, USA). Experiments were performed during peak disease activity (DSS_A_) 10 days after the start of DSS treatment and during remission (DSS_R_) when the disease activity index had returned to baseline (21 days after DSS treatment), as previously described [9,13]. To suppress colitis induced AngII signaling, mice were treated with the ACE inhibitor Perindopril (i(ACE)). Perindopril treatment (i(ACE), intraperitoneal (ip): 3 mg/kg/day; Sellek Chemicals, Huston, TX, USA) started on day 5 of DSS treatment and continued until sacrifice (DSS_i(ACE)_) on day 10 [9,14]. All animal procedures were performed with the approval of the IACUC of Rush University and in accordance with the National Institute of Health’s Guide for the Care and Use of Laboratory Animals (IACUC Protocol number: #25-035).

### 2.2. Isolation of Mouse Ventricular Myocytes

Mouse ventricular myocytes (VM) were isolated by enzymatic digestion using Langendorff perfusion (Harvard Apparatus, Holliston, MA, USA), as previously described [15,16]. Afterwards, the ventricles were dissected, cut into strips, and incubated in digestion buffer (mg/L): 0.1 Liberase TM (Roche, Indianapolis, IN, USA), 0.14 trypsin (Thermo Fisher Scientific, Waltham, MA, USA), 1 Protease type XIV (Sigma-Aldrich Inc., St. Louis, MO, USA) for 20 min at 37 °C. Digestion was stopped by addition of bovine calf serum (Hyclone, Thermo Fisher Scientific, Waltham, MA, USA) before Ca^2+^ was reintroduced to the solution in a stepwise manner till final [Ca^2+^] of 1.5 mM was reached [9,17].

### 2.3. Calcium Imaging and Cell Shortening

Ventricular excitation–contraction coupling (E-CC) was assessed in isolated VMs by cell shortening using a IonOptix MyoCam-S video camera, the IonWizard 7.3 data acquisition and analysis software (Ionoptix, Milton, MA, USA). Fluorescent measurements of [Ca^2+^]_i_ were performed with Fluo-4/AM (10 μmol/L; Thermo Fisher Scientific, Waltham, MA, USA) using line scanning confocal imaging (A1 Nikon Instruments Inc., Melville, NY, USA). Fluorescence was excited at 485 nm and emission collected at 515 nm using a photomultiplier tube (Nikon Instruments Inc., Melville, NY, USA).

Line scan images were obtained by confocal microscopy (Nikon A1R, Nikon Instruments Inc., Melville, NY, USA) at 425 lines/s with the line positioned across the width of the cell. Data were analyzed and quantified in ImageJ version 1.54 (U.S. National Institutes of Health, Bethesda, MD, USA). Changes in [Ca^2+^]_i_ are presented as changes in fluorescence (ΔF) of background-subtracted fluorescence signals, normalized to the resting fluorescence (F_0_). To obtain the coefficient of variation (CoV = mean/standard deviation of the CaT amplitudes) of the Ca release across the width of the cell, the variability of the local CaT amplitude was quantified at 20 ms after stimulation.

### 2.4. T-Tubular Staining

To determine the t-tubular density, plasma membrane of isolated VMs from CTL, DSS_A_, DSS_R_, and DSS_i(ACE)_ hearts was stained with di-8-ANEPPS (5 µmol/L, 20 min; Thermo Fisher Scientific, Waltham, MA, USA). After washout, cells were plated onto laminin (1 mg/mL)-coated coverslips. Using confocal microscopy, images of the cells were obtained (excitation: 468 nm/emission: 635 nm; Nikon A1R, Nikon Instruments Inc., Melville, NY, USA) and t-tubular density was quantified in ImageJ version 1.54 (U.S. National Institutes of Health, Bethesda, MD, USA) using binary density calculations (thresholding) and is presented as the percentage of cell surface occupied by stained t-tubular structures.

### 2.5. Autophagy Assay

Isolated VMs from CTL, DSS_A_, DSS_R_, and DSS_i(ACE)_ hearts were stained according to the manufacturer’s instructions, with CYTO-ID^®^, a dye that selectively accumulates in autophagic vacuoles and Hoechst33342 for nuclear staining (ENZ-51031, Enzo Life Science, Farmingdale, NY, USA). Using confocal microscopy (Nikon A1R, Nikon Instruments Inc., Melville, NY, USA) images of the autophagic vacuoles and nuclear staining were obtained (excitation: ~499 nm/emission: 548 nm for CYTO-ID^®^, and excitation 350 nm/emission 461 nm for Hoechst33342). To ensure reproducibility, recordings were made at the depth where the nucleus was fully visible. The degree of autophagy was quantified as the number of autophagic vesicles per cell.

### 2.6. Western Blotting

Left ventricular tissue was collected, frozen in liquid nitrogen, and lysed with hot 1x Laemmeli buffer: 5% SDS, 10% glycerol, 62.5 mM Tris-HCl (pH 6.8; Sigma-Aldrich Inc., St. Louis, MO, USA). Protein lysates were quantified using a BCA assay kit (Thermo Fisher Scientific, Waltham, MA, USA). Protein (30–40 μg) was loaded onto 4–20% tris–glycine gel for SDS-PAGE (Novex, Thermo Fisher Scientific, Waltham, MA, USA). After protein transfer, membranes were stained with Ponceau S for total protein quantification, and primary antibody incubation was subsequently performed overnight (JPH-2: 1:2000; 40-5300; Calpain1: 1:1000, #MA1-12434; Thermo Fisher Scientific, Waltham, MA, USA; GAPDH: 1:75000; D16H11, Calpain-2: CAPN2: 1:1000; #2539; LC3B: 1:1000, #2775; Cell Signaling Technology, Danvers, MA, USA). Species-specific horseradish peroxidase-conjugated secondary antibodies were used (1:20,000), and visualization was accomplished by Western Pierce^TM^ enhanced chemiluminescence (Thermo Fisher Scientific, Waltham, MA, USA). The Syngene PXi 6 imager in combination with the GeneSYS software 1.9.0 (Syngene International Ltd., Baltimore, MD, USA) was used for image acquisition. Changes in protein level were quantified with ImageJ version 1.54 (U.S. National Institutes of Health, Bethesda, MD, USA).

### 2.7. Statistics

A Shapiro–Wilk test was performed to assess the normal distribution of the data. Comparisons are made using a Student’s *t*-test (2 data groups) or one-way ANOVA (>3 data groups) with Tukey’s multiple comparison test. When the Shapiro–Wilk test re-vealed a non-parametric distribution, the one-way ANOVA on ranks, or Mann–Whitney U test was used as indicated. Data are presented when possible as scatter plots, as mean ± standard deviation (SD). The sample size is provided as number of cells/number of hearts. Throughout the manuscript, the level of significance was set at *p* < 0.05. Confidence intervals and post hoc power analysis (G*Power, version 3.1.9.6, Heinrich Heine Universität Düsseldorf, Germany) are provided in the supplement for all single-cell experiments.

Generative artificial intelligence (GenAI) has not been used to generate any part (experiments, text, data, graphics, or interpretation) of this study.

## 3. Results

### 3.1. Active Colitis Attenuates Ventricular Contractile Strength

**Figure 1:**
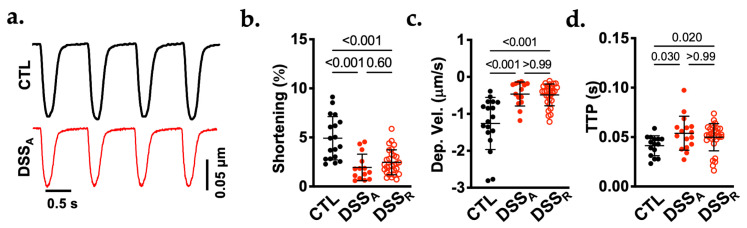
Colitis attenuates VM contractile function: (**a**). Representative traces of sarcomere shortening recorded in VMs from CTL and DSS_A_ mice. Quantification of (**b**) sarcomere shortening, (**c**) shortening velocity, and (**d**) time to peak (TTP) shortening of isolated VMs from CTL (cells/mice: 18/3), DSS_A_ (14/4), and DSS_R_ (29/4) mice. Data are presented as individual measurements and mean ± SD. Significance was determined by Kruskal–Wallis multiple comparisons test. Confidence interval and power calculation provided in Supplementary Table S1.

To determine if active colitis leads to alterations in ventricular contractile function, we used a mouse model of DSS (3.5% *w*/*v*, 7 d)-induced active colitis [9,13]. Experiments were performed under control (CTL) conditions, during active colitis (DSS_A_), and remission (DSS_R_). Changes in the contractile function were quantified by sarcomere shortening in field-stimulated (1 Hz) VMs. During active colitis, VMs exhibited attenuated sarcomere shortening (Figure 1a,b), slower shortening velocity (Figure 1c) and increased time to peak (TTP; Figure 1d). These changes were not fully reversible after 21 days of recovery. Our results are consistent with observations that patients with active colitis exhibit attenuated ventricular contractile strength and increased strain [18,19].

**Figure 2:**
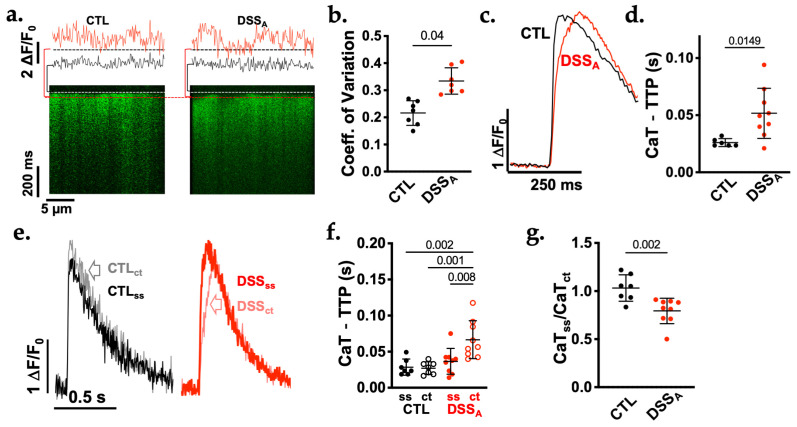
Colitis delays CaT upstroke velocity and increases heterogeneity of Ca release in VMs: (**a**). Representative line scans (bottom) and ΔF/F_0_ plots (top) from CTL and DSS_A_ myocytes across the width of the cell. ΔF/F_0_ plots shown (top) were obtained immediately before (black) and 20 ms after (red) stimulation of CaT. The time points are indicated by brackets (left of line scan). (**b**). Quantification of the coefficient of variation 20 ms after the stimulus in CTL (*n* = 7/2) and DSS_A_ (*n* = 7/2) VMs. (**c**). ΔF/F_0_ plots from CTL (black) and DSS_A_ (red) myocytes obtained from the width of the cell. (**d**). Quantification of CaT-time to peak (TTP) in CTL (black, *n* = 6/2) and DSS_A_ (red, *n* = 9/2) VMs across the width of the cell. (**e**). Representative CaTs from CTL and DSS_A_ myocytes obtained at the sub-sarcolemmal (CaT_ss_) and central (CaT_ct_, open arrow) area of the cell and (**f**) quantification of Ca-TTP (CTL: *n* = 7/2; DSS_A_: *n* = 9/2) at those locations. (**g**). Quantification of the CaT amplitude as the normalized ratio of CaT_ss_ vs. CaT_ct_ in CTL (7/2) and DSS_A_ (9/2) VMs. Data are presented as individual measurements and mean ± SD. Significance was determined by Tuckey multiple comparison (**f**) or Student *t*-test (**b**,**d**,**g**). Confidence interval and power calculation provided in Supplementary Table S2.

To determine if the attenuated cellular shortening is a consequence of an altered kinetics of E-CC, we quantified the cellular Ca^2+^ handling properties using line-scanning confocal microscopy [16]. In VMs, the rapid rise in the CaT relies on the t-tubular system [4]. To assess alterations in the homogeneity of CICR throughout the VM, the scan line was positioned across the width of the cell. ΔF/F_0_ plots obtained at the onset of CaT (20 ms after stimulation) revealed that the rise in [Ca^2+^]_i_ along the scan line was homogeneous in CTL myocytes, while during active colitis, the cell displayed delays in Ca^2+^ release (Figure 2a). The variability in subcellular Ca^2+^ release was quantified as the coefficient of variation (CoV; 2.3.) of the CaT amplitude. At 20 ms after stimulation CoV was increased during active colitis (Figure 2b). To determine how the altered Ca^2+^ release impacts the kinetic of CaT, ΔF/F_0_ was plotted against time. Quantification of [Ca^2+^]_i_ across the entire width of the cell revealed an increased time to peak (TTP) of the CaT during active colitis (Figure 2c,d) [9]. To assess if the delayed activation of central release sites is the cause for the attenuation of the CaT rise time, CaT-TTP was quantified in a spatially defined manner at the sub-sarcolemmal (CaT_ss_) and central (CaT_ct_) regions of the VMs (Figure 2e). No significant difference in TTP was determined between CaT_ss_ and CaT_ct_ regions of CTL VMs supporting a homogeneous rise of [Ca^2+^]_i_ throughout the cells (Figure 2e,f). The normalized difference between CaT_ss_ vs. CaT_ct_ amplitude in CTL cells (CaT_ss_/CaT_ct_: 1.03 ± 0.12; Figure 2g) further indicates that the CaT amplitude is comparable throughout the cell. During active colitis, there was no difference in CaT-TTP between CaT_ss_ in CTL and DSS VMs, advocating for an unchanged E-CC system at the sub-sarcolemmal membrane. However, CaT-TTP at CaT_ct_ in DSS VMs was prolonged compared to DSS CaT_ss_ and all other release sites in CTL VMs (Figure 2e,f). In addition, the ratio of the CaT amplitude CaT_ss_/CaT_ct_ (0.83 ± 0.07; Figure 2g) was reduced compared to CTL, indicating a reduced CaT amplitude in the central regions of these myocytes.

### 3.2. Increased Inhomogeneity of Ventricular Ca^2+^ Release Units During Colitis

**Figure 3:**
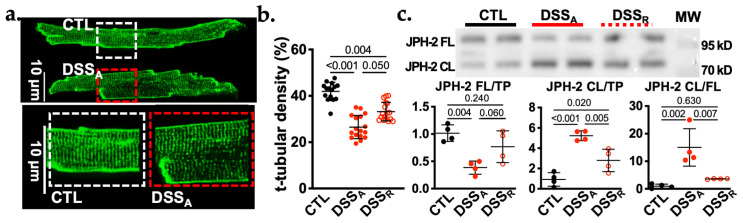
Colitis induces reversible t-tubular remodeling in ventricular myocytes. (**a**). Representative images of di-8-ANNEPS stained CTL and DSS_A_ myocytes. Dashed squares indicate area magnified below. (**b**). Quantification of the t-tubular density in CLT (15/2), DSS_A_ (17/3) and DSS_R_ (17/2) myocytes. (**c**). Western blot image (top) of ventricular protein lysate from CTL (*n* = 4), DSS_A_ (*n* = 4), and DSS_R_ (*n* = 4) mice stained for JPH-2 and quantification of the same blot (bottom). Full length (FL: 90 kDa) and cleaved (CL: >70 kDa) JPH-2 bands were normalized to total protein obtained by Ponceau-S staining (Supplementary Figure S1) and quantified individually or as the ratio of JPH-2 CL/FL (right). Data are presented as mean ± SD. Significance was determined by one-way ANOVA and Tuckey’s multiple comparisons test. Confidence interval and power calculation provided in Supplementary Table S3.

A dissolution of Ca^2+^ release units can be the consequence of a loss of the t-tubular system [4]. To quantify potential changes in the t-tubular structure during active colitis, the plasma-membranes of isolated VMs were stained with di-8-ANEPPS [15]. In CTL myocytes, t-tubules were homogeneously distributed throughout the cytoplasm, while during active colitis, VMs exhibited areas within the cytoplasm that were void of membrane staining (Figure 3a). Quantification revealed a significant reduction in the VMs t-tubular density during active colitis (Figure 3b). The reduced t-tubular density tended to be restored upon remission. The experimental results indicate that the colitis-induced loss of the t-tubular system contributes to an inhomogeneous and delayed rise in [Ca^2+^]_i_.

### 3.3. Colitis Induced Cleavage of Junctophilin-2

A reduced t-tubular density has been linked to attenuated expression and/or cleavage of Junctophilin 2 (JPH-2) [4,8]. Western blot analysis of protein lysates from CTL, DSS_A_, and DSS_R_ ventricles revealed two JPH-2 protein bands in all samples. The bands were consistent with the molecular weight (MW) of the full-length (FL) protein at 90 kDa and with a cleaved (CL) JPH-2 protein fraction above 70 kDa (Figure 3c, top) were determined. The density of the band representing JPH-2 FL was significantly reduced in DSS_A_ myocytes compared to those from CTL mice, whereas the band representing JPH-2 CL dominated in myocytes from DSS_A_ animals (Figure 3c, bottom). During remission, JPH-2 CL and the ratio of JPH-2 CL/FL significantly recovered compared to active colitis, and JPH-2 CL/FL even returned to CTL levels.

### 3.4. AngII Signaling During Active Colitis

**Figure 4:**
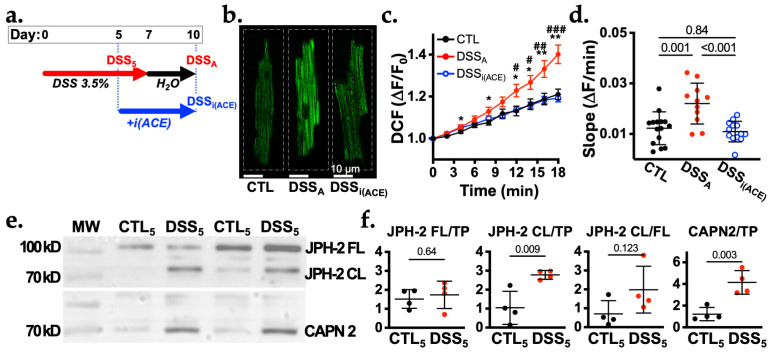
Colitis induces AngII dependent increase in ROS production. (**a**). Schematic representation of the treatment protocol for DSS-induced colitis. Experimental days for active colitis (DSS_A_), start of ACE inhibitor (i(ACE): ip: 3 mg/kg/day) treatment (day 5 of DSS treatment: DSS_5_), and end of i(ACE) treatment (DSS_i(ACE)_) are marked. (**b**). Representative images of CTL, DSS_A_, and DSS_i(ACE)_ VMs stained with DCF. (**c**). Average DCF fluorescence over time recorded in CTL (*n* = 14/2), DSS_A_ (*n* = 11/2) and DSS_i(ACE)_ (*n* = 13/2) myocytes. (**d**). Quantification of the change of DCF fluorescence over time (slope) for CTL (*n* = 14/2 VMs/hearts), DSS_A_ (*n* = 11/2) and DSS_i(ACE)_ (*n* = 13/2) VMs. (**e**). Western blot image of ventricular protein lysate from CTL and DSS treated mice after 5 days of DSS treatment (CTL_5_, DSS_5_) stained for JPH-2 and CAPN2. (**f**). Quantification of the Western blots shown for full length (FL: 90 kDa), cleaved (CL: 70 kDa) JPH-2 (left), and CAPN2 (right) normalized to total protein (TP) obtained by Ponceau-S staining (Supplementary Figure S2). The ratio of CL to FL JPH-2 (JPH-2 CL/FL) reflects the shift toward cleaved JPH-2 during colitis. Data are presented as mean ± SD. Significance was determined by Tuckey’s multiple comparisons ((**c**,**d**); CTL vs. DSS: * p < 0.05, ** p < 0.01; and DSS vs. DSS_i(ACE)_: ^#^ *p* < 0.05, ^##^ *p* < 0.01, ^###^ *p* < 0.001) or Student’s *t*-test (**f**). Confidence interval and power calculation for (**d**) provided in Supplementary Table S4.

We and others have demonstrated that AngII levels are increased in the serum of animal models and patients with active colitis [9,13,20]. To determine if AngII signaling contributes to the colitis-induced ventricular t-tubular remodeling we exposed mice to DSS and, upon the onset of weight loss and increase in disease activity (d5), mice were treated with the ACE inhibitor Perindopril (i(ACE): 3 mg/kg/day) until the day of sacrifice (d10, DSS_i(ACE)_; Figure 4a) [9,13].

It is known that activation of AT1R can increase ROS production in cardiac myocytes [21]. To determine the impact of AngII during active colitis, we assessed cellular ROS production in CTL, DSS_A_, and DSS_i(ACE)_ VMs, quantifying changes in DCF fluorescence over time. The time-dependent rise in fluorescence revealed an increased ROS production in DSS_A_ compared to CTL VMs that was prevented in DSS_i(ACE)_ mice (Figure 4b–d). The experiments support our prior findings [9,13] of a colitis induced increase in ventricular AngII signaling that can be reversed by i(ACE) treatment.

### 3.5. AngII Signaling Contributes to T-Tubular Remodeling

To determine if i(ACE) can prevent the colitis induced t-tubular remodeling, we assessed the status of JPH-2 expression and cleavage at the start of i(ACE) treatment (day 5 of DSS treatment (DSS_5_), Figure 4a). Western blot analysis of ventricular tissue lysates (CTL_5_, DSS_5_) confirmed that at day 5 of DSS treatment, JPH-2 cleavage was already significantly increased (Figure 4e,f). The elevated levels of JPH-2 CL in DSS_5_ mice coincided with heightened protein levels of calpain-2 (CAPN2), a protein shown to cleave JPH-2 into a <75 kDa and a ~20 kDa fraction [22,23]. Calpain 1 expression on the other hand remained unchanged (Supplementary Figure S3) during active colitis. The results suggest that the destabilization of the Ca^2+^ release unit potentially through CAPN2-dependent JPH2 cleavage was already initiated at the onset of i(ACE) treatment.

**Figure 5:**
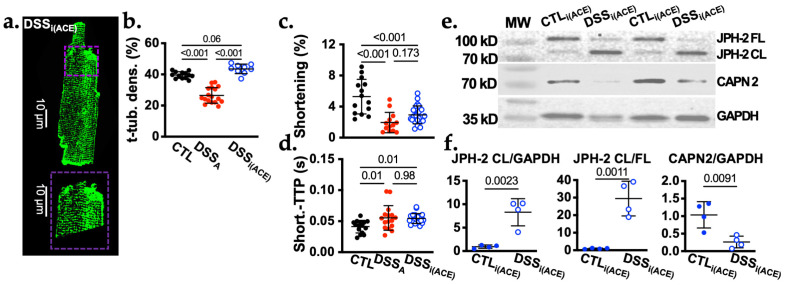
Colitis induced t-tubular remodeling can be prevented by ACE inhibition. (**a**). Representative image of a di-8-ANNEPS stained DSS_i(ACE)_ myocyte (dashed square indicates area magnified below) and quantification of (**b**) the t-tubular density in VMs isolated from CLT, DSS_A_, and DSS_A_ mice treated with i(ACE) (*n* = CLT: 15/2; DSS_A_: 17/3 and DSS_i(ACE)_: 9/2). (**c**). Sarcomere shortening (*n* = CTL: 18/3; DSS_A_: 16/4; DSS_i(ACE)_: 23/4), and (**d**) shortening- time to peak (TTP) in CTL (*n* = 15/2), DSS_A_ (*n* = 16/4), and DSS_i(ACE)_ (*n* = 21/3) VMs. (**e**). Western blot image of ventricular protein lysate from CTL_i(ACE)_ and DSS_i(ACE)_ mice stained for JPH-2, CAPN2, and GAPDH. (**f**). Quantification of the Western blots shown for cleaved (CL: 70 kDa) JPH-2 (left), the ratio of CL to full length (FL: 90 kDa) JPH-2, and CAPN2 (right) normalized to GAPDH (Original blots provided in Supplementary Figure S4). Data are presented as mean ± SD. Significance was determined by Tuckey’s multiple comparisons test (**b**–**d**) or Student’s *t*-test (**e**,**f**). Confidence interval and power calculation for (**b**–**d**) provided in Supplementary Table S5.

Quantification of the t-tubular density in isolated VMs from CTL and DSS_A_ animals treated with i(ACE) (CTL_i(ACE)_, DSS_i(ACE)_) revealed that i(ACE) preserved the t-tubular structure during active colitis (Figure 5a,b). Despite the normalization of the CaT (see [9]), cell shortening and shortening-TTP (Figure 5c,d) were not restored in i(ACE)-treated animals suggesting that in addition to Ca^2+^ cycling additional mechanisms contribute to the attenuation of contractile strength during colitis.

### 3.6. AngII Dependent Activation of Autophagy

**Figure 6:**
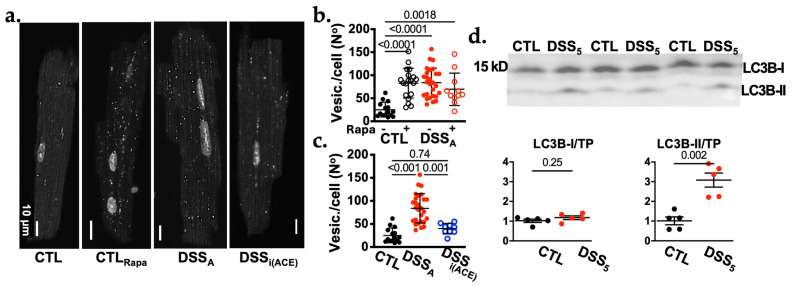
Autophagy is activated in VMs during active colitis. (**a**). Representative image of CTL and DSS_A_ myocytes stained for autophagic vesicles. (**b**). Quantification of the number of autophagic vesicles from CLT (*n* = 15/2, VMs/mice) and DSS_A_ (*n* = 27/2) myocytes before and after (CTL: *n* = 19/2; DSS_A_: *n* = 11/2) treatment with rapamycin. (**c**). Number of autophagic vesicles per cell in CLT, DSS_A_ and DSS_i(ACE)_ (*n* = 9/1) myocytes. (**d**). Western blot image (top) of ventricular protein lysate from CTL (*n* = 5) and DSS_5_ (*n* = 5) mice after 5 days of H_2_O or DSS treatment, respectively stained for LC3B. The quantification of LC3B-I (upper band) and LC3B-II (lower band) normalized to total protein (TP) is shown below. TP was obtained by Ponceau-S staining (Supplementary Figure S5). Data are presented as mean ± SD and significance was determined by Tuckey’s multiple comparisons test. Confidence interval and power calculation for (**b**,**c**) provided in Supplementary Table S6.

Besides a decrease in JPH-2 FL, cellular autophagy plays a key role in t-tubular maintenance and remodeling [24,25], and autophagic marker proteins were shown to be upregulated after t-tubular damage and during t-tubular degradation [24,26]. We quantified the presence of autophagic vesicles in VMs isolated from CTL, DSS_A_, and DSS_i(ACE)_ hearts to determine if changes in autophagy correlate with the t-tubular remodeling. For this purpose, isolated VMs were stained with a marker for autophagic vesicles and the number of vesicles per cell was quantified (Figure 6a–c). In freshly isolated VMs, the number of autophagic vesicles was significantly increased in DSS_A_ compared to CTL myocytes (Figure 6a,b). Treatment of cells with Rapamycin (50 nmol/L, 1 h), an mTOR inhibitor and autophagy inducer [27], significantly increased the number of autophagic vesicles in CTL, but not DSS_A_ myocytes suggesting that autophagy was already increased (Figure 6b). The latter was further supported by immunoblots of microtubule-associated protein 1A/1B-light chain 3 (LC3B). The autophagosome-bound isoform (LC3B-II) which derives from the cytosolic LC3B-I, was increased during active colitis (Figure 6d). Treatment of mice with i(ACE) (Figure 4a) prevented the colitis induced increase in ventricular autophagy (Figure 6c). We therefore propose that despite the persisting JPH-2 cleavage in DSS_i(ACE)_ VMs, i(ACE) preserves the t-tubular structure by attenuating their autophagic degradation.

## 4. Discussion

In the present study we demonstrate for the first time that active colitis causes a reversible reduction in ventricular t-tubular density potentially due to CAPN2 induced JPH-2 cleavage and autophagy-mediated t-tubular degradation. These processes can be prevented by the suppression of AngII signaling through ACE inhibition. However, the preservation of t-tubules during colitis does not completely restore contractility of VMs suggesting additional colitis-induced mechanisms affect cell shortening.

### 4.1. Colitis-Induced Changes in Cardiac Function

Colitis induces ventricular remodeling in patients as well as animal models. In patients, electrophysiological changes such as increased QT duration and dispersion [28,29], as well as decreased contractile strength and increased strain were described [18]. The latter is most prominent during the active phase of the disease. Mouse models of active colitis replicate the clinically observed changes in ventricular electrophysiology [9]. In addition, we have demonstrated altered cellular Ca^2+^ handling properties which increase propensity for cellular and tissue alternans in the ventricular muscle [9]. Alternans enhances the dispersion of repolarization and increases the risk for ventricular arrhythmia. In the current study we demonstrate that in the mouse model of DSS-induced active colitis, VMs exhibit attenuated shortening, which can be partially explained by a delayed rise of [Ca^2+^]_i_. Consistent with human data, these changes were mostly reversible upon remission, making this mouse model a good tool to study colitis-induced changes in cardiac function.

### 4.2. Active Colitis Attenuates the Rise Time of the Ca^2+^ Transient and Cell Shortening

In VMs the CaT depends on the amount of Ca^2+^ entering the cell through LCC, the amount of Ca^2+^ released from the SR, as well as the kinetics of Ca^2+^-influx and release. The more homogeneous the rise in [Ca^2+^]_i_ throughout the cell, the faster the CaT upstroke velocity and the higher the CaT amplitude and [Ca^2+^]_i_ available for contraction [4,30,31,32]. LCCs are prominently localized to the t-tubular structure that in VMs extends throughout the cytoplasm in transverse and longitudinal direction [33,34]. The AP propagating along the t-tubular system allows for voltage-dependent Ca^2+^-entry not only in the periphery, but also in the depth of the cell. The Ca^2+^-release unit enables Ca^2+^-influx induced CICR and a rapid and spatially homogeneous rise of [Ca^2+^]_i_ even in the center of the cell. The latter can be seen in CTL mouse VMs where line scan images do not show significant differences in the CaT-TTP between the sub-sarcolemmal space and the center of the myocyte (Figure 2d–g).

The subcellular homogeneity of the ventricular Ca^2+^ release can become disrupted under physiological conditions as a consequence of unloading and aging [35], and under pathophysiological conditions such as heart failure [36], dilated cardiomyopathy [37], or myocardial infarction [32]. In these cases, an increased subcellular heterogeneity of the CICR, an increased CaT-TTP, reduced CaT amplitude, and an attenuated contractile strength [4,8] have been linked to the alterations in t-tubular density. The loss of t-tubules also decreases plasma membrane surface area and can affect the cellular electrophysiological properties by altering current density as well as kinetics of LCC, NCX and Na-K ATP-ases [34,38]. In our previous study we did not detect changes in the ventricular AP [9], suggesting that the t-tubular remodeling during colitis does not induce extensive ion channel remodeling [33]. This is also consistent with our finding that the TTP of the sub-sarcolemmal CaT is comparable between DSS_A_ and CTL myocytes (Figure 2f).

The most critical impact of a reduced t-tubular density lies in the alterations of the Ca^2+^ release units and their subcellular distribution. With the loss of t-tubules, AP induced CICR remains more restricted to the sub-sarcolemmal regions of the cell [39]. As a consequence, CaT rise throughout the cell becomes more comparable to that of atrial cells that are, especially in smaller species, mostly void of a t-tubular system. In atrial as well as de-tubulated cells, central Ca^2+^ release depends on the fire–diffuse–fire mechanism where sub-sarcolemmal Ca^2+^ release diffuses toward the central regions of the cell, amplified by CICR along the way [15,30,33]. Consequently, the rise time of the central CaT and the whole cell CaT is prolonged. During active colitis the increased delay in whole cell CaT-TTP, the increased CoV at the onset of CaT, and the reduction in CaT_ss_/CaT_ct_ are consistent with the observed attenuation of the t-tubular density.

### 4.3. Mechanism of T-Tubular Remodeling During Colitis

The structural integrity and maintenance of t-tubules encompass structural proteins like JPH-2, amphiphysin (BIN1), ankyrin, caveolin and telethonin but also phospholipids and cytoskeletal proteins such as microtubules [40]. JPH-2 with its two transmembrane domains, anchors the sarcoplasmic reticulum to the plasma membrane. Through protein biding, it maintains the structural integrity of the Ca^2+^ release unit. Loss of JPH-2 results in hypertrophic cardiomyopathy, hyperactive RyRs [6,26], loss of Ca^2+^ release micro-domains [41], as well as de-tubulation [42]. JPH-2 mutations have been linked to hypertrophic, dilated, and arrhythmic cardiomyopathy [43,44]. Our data demonstrate a colitis- induced decrease in full length JPH-2, supporting the view that the loss of functional protein contributes to t-tubular instability.

Loss of cardiac JPH-2 has been linked to mRNA dependent downregulation, micro-tubule mediated redistribution, as well as calpain 1/2 (CAPN1/2) dependent cleavage [45]. Increased CAPN activity results in JPH-2 cleavage into >70 and ~25 kDa fragments. The lower molecular weight fragments can translocate to the nuclear envelope and affect transcriptional regulation of cardiac hypertrophy by blocking MEF2 expression [23]. CAPN1 is activated at lower [Ca]_i_ than CAPN2, while CAPN2 activation can also occur through ERK1/2 dependent phosphorylation as a consequence of cytokine as well as AngII signaling [46]. Since there are no indications of elevated [Ca]_i_ in ventricular tissue during active colitis, we suggest that JPH-2 cleavage may result from increased CAPN2 activity. This is consistent with an increase in CAPN2 expression as early as day 5 after colitis induction, and the attenuation of CAPN2 protein levels when ACE is inhibited during colitis. Additionally, the lack of an increase in CAPN1 (Supplementary Figure S3) indicates that CAPN2 contributes to JPH-2 degradation. JPH-2 remodeling in heart failure and mice with muscular dystrophy was also linked to an increased myofilament density which results in JPH-2 mislocalization and t-tubular degradation [47]. However, myofilament proteolysis due to increased calpain activity would be expected to attenuate any microtubule dependent t-tubular remodeling.

We have previously demonstrated that AngII levels are increased during active colitis. Ventricular alternans and atrial remodeling induced by active colitis could be attenuated through ACE or AT1R inhibition [9,13]. AngII activates AT1R dependent G_q_ signaling and among other effects, promotes an increase in NOX2-dependent ROS production [16,48]. In the absence of [Ca^2+^]_i_ overload, the oxidative stress induced by AngII can activate transcription factors, e.g., NFkB, that can increase CAPN2 expression [49]. Consistent with previously published findings, our new data further supports increased AngII signaling during active colitis, demonstrating ACE-dependent ROS production, CAPN2 upregulation, and t-tubular remodeling, as well as an increased accumulation of autophagic vesicles and increase in LC3B-II (Figure 6). CAPN2 protein levels dropped below CTL levels in DSS_i(ACE)_ animals (Figure 5e,f). Since CAPN2 was already increased at the onset of i(ACE) treatment, this decrease is likely the consequence of an autoregulatory mechanism, where activated CAPN2 self-degrades over time [50].

### 4.4. Recovery of the T-Tubular Structure

Studies show that t-tubular remodeling induced by heart failure or dilated cardiomyopathy can be reversed after normalization of load, re-synchronization therapy, or normalization of intracellular Ca^2+^ handling through SERCA overexpression [51]. Also, JPH-2 overexpression was sufficient to restore t-tubular remodeling in JPH-2 KO mice [8,52]. Nevertheless, the knowledge on the proteins required for t-tubular recovery is still lacking. Besides JPH-2, the overexpression of amphiphysin-2 (BIN1), the phosphoinositide phosphatase (MTM1), or dysferlin were shown to recover disease-induced changes in the t-tubular structure [8]. Our data show that i(ACE) treatment prevents the loss of the t-tubular structure during active colitis by the attenuation of CAPN2 expression (Figure 5e). Interestingly, i(ACE) did not restore full length JPH-2, suggesting that the t-tubular structure can be maintained or restored despite JPH-2 cleavage. Another mechanism that is critically involved in t-tubular remodeling is an autophagy. Evidence for the importance of autophagy in t-tubular remodeling stem from an animal model of dilated cardiomyopathy, where gaps in the t-tubular structure coincided with locations of increased lysosomal density [26], and from drosophila studies, where autophagy is required for t-tubular disassembly [24]. AngII-induced ROS production increases cellular autophagy [53,54]; however, CAPN2 also increases the autophagic flux [55]. We started i(ACE) treatment when animals already exhibited signs of active colitis. CAPN2 was already increased and JPH-2 cleaved, suggesting that t-tubular remodeling was initiated early in disease progression. At this point, we cannot fully distinguish if i(ACE) only prevents the progression of t-tubular remodeling or also promotes the recovery of the t-tubular structure. The lack of i(ACE)-dependent restoration of full length JPH-2 would support the view that t-tubules and the Ca^2+^ release unit are still destabilized. The i(ACE)-dependent reduction in ROS, CAPN2 protein, and autophagy, however, advocates for a prevention of the t-tubular degradation despite a JPH-2 cleavage-dependent destabilization of the Ca^2+^ release unit.

### 4.5. Limitations

We demonstrate that the t-tubular structure and Ca-handling properties can be restored/maintained when mice are treated with i(ACE) during the induction of colitis. At the same time point the sarcomere shortening remains attenuated and does not fully recover during remission (Figure 1b–d and Figure 5c,d). These data would suggest that attenuated myofilament function is more persistent than the change in Ca^2+^ handling. This may be because calpain-dependent proteolysis of contractile filaments needs to be compensated by increased protein expression. Alternatively, inflammation was shown to attenuate the myofilament Ca-sensitivity as a consequence of increased ROS or nitric oxide signaling. Also, inflammation-induced mitochondrial dysfunction may not only further increase cellular ROS production but also leads to a depletion of cellular ATP, thereby attenuating myofilament function. The mechanism of altered myofilament function will be investigated in future studies.

The mouse model of DSS-induced colitis closely reproduces acute, chronic, and relapsing phases of the disease, and the induced dysplasia resembles the clinical phenotype of human colitis. Cytokines (IL-6, -16, and -22) and chemokines (CCL2, CCL3, and CXCL1) related to human colitis are also upregulated. However, alternative models of colitis will be employed in future studies to confirm ventricular remodeling observed in this study is independent from the model of colitis. Also, in this study we only assessed the impact of colitis on cardiac remodeling in male mice. Up to the age of 45, men and women exhibit the same incidence for Ulcerative colitis, while afterwards men have a 20% higher risk than women. Consistent with patient data, male mice exhibit more severe colitis than female mice. To assess sex-specific differences in colitis-induced cardiac remodeling in the future, the model has to be adjusted to generate comparable disease activity in male and female mice.

## 5. Conclusions

We demonstrate that active colitis promotes t-tubular remodeling by AngII-dependent CAPN2 activation. The resulting attenuation of CaT amplitude and kinetics are reversible upon remission and can be prevented by attenuation of AngII signaling during active colitis. The experimental results suggest that patients with active colitis especially those with high blood pressure would benefit from treatment with AT1R blockers or ACE inhibitors to prevent colitis-induced changes in cardiovascular function.

## Data Availability

The data that support the findings of this study are available from the corresponding author upon reasonable request.

## Supplementary Materials

The following supporting information can be downloaded at https://www.mdpi.com/article/10.3390/biom16040503/s1, Table S1: Confidence interval and power calculation for experiments in Figure 1b–d; Table S2: Confidence interval and power calculation for experiments in Figure 2b,d,g,f; Table S3: Confidence interval and power calculation for experiments in Figure 3b; Figure S1: Original blots shown in Figure 3c; Table S4: Confidence interval and power calculation for experiments in Figure 4d; Figure S2: Original blots shown in Figure 4e; Figure S3: Original blots showing protein levels of calpain 1 during active colitis; Table S5: Confidence interval and power calculation for experiments in Figure 5b–d; Figure S4: Original blots shown in Figure 5e; Table S6: Confidence interval and power calculation for experiments in Figure 6b,c; Figure S5: Original blots shown in Figure 6d.

## Abbreviations

The following abbreviations are used in this manuscript:

ACE	Angiotensin-converting enzyme
AngII	Angiotensin II
AP	Action potential
APD	Action potential duration
AT1R	Angiotensin II receptor type 1
CaT	Calcium transient
CAPN2	Calpain 2
CICR	Ca-induced Ca-release
DAI	Disease activity index
DSS	Dextran sulfate sodium
i(ACE)	ACE inhibitor
IBD	Inflammatory bowel disease
IL	Interleukin
JPH-2	Junctophilin 2
LCC	L-type Ca channel
RAS	Renin-angiotensin system
TTP	Time to peak
UC	Ulcerative colitis
