# Supplementary material for: Active Colitis Attenuates Ventricular Excitation–Contraction Coupling by T-Tubular Remodeling
Source: Biomolecules. 2026 Mar 27;16(4):503. doi: 10.3390/biom16040503 (PMC13113157; doi:10.3390/biom16040503)
Supplement: Supplementary file 1 [file biomolecules-16-00503-s001.zip › biomolecules-4177732-supplementary.pdf]

Supplementary Table S1			CTL	DSS	DSS_R
Figure 1	1b	Lower 95% CI	0.077	0.027	0.041
		Upper 95% CI	0.122	0.087	0.068
		N=61, α = 0.05, f = 0.46, power > 90%			
	1c	Lower 95% CI	53.73	39.15	44.34
		Upper 95% CI	73.11	49.13	55.06
		N=61, α = 0.05, f = 0.46, power > 95%			
	1d	Lower 95% CI	0.035	0.044	0.044
		Upper 95% CI	0.046	0.063	0.054
		N=61, α = 0.05, f =11.9, power > 90%			

Supplementary Table S1: Confidence interval (CI) and power calculation (one-way ANOVA) for single cell experiments shown in Figure 1. Provided are sample size (N), significance level (α), effect size (f), and power.

Supplementary Table S2			CTL	DSS		
Figure 2	2b	Lower 95% CI	0.174	0.289		
		Upper 95% CI	0.257	0.379		
		N=14, α = 0.05, d = 2.6, power > 95%				
	2d	Lower 95% CI	0.022	0.034		
		Upper 95% CI	0.029	0.068		
		N=14, α = 0.05, d = 1.6, power > 85%				
	2g	Lower 95% CI	0.904	0.692		
		Upper 95% CI	1.160	0.896		
		N=16, α = 0.05, d = 1.8, power > 90%				
	2f		CTL_{ss}	CTL_{ct}	DSS_{ss}	DSS_{ct}
		Lower 95% CI	0.018	0.019	0.022	0.0464
		Upper 95% CI	0.038	0.035	0.050	0.0867
		CTL_{ct} vs. DSS_{ct}: N=36, α = 0.05, f = 1.5, power > 95%				

Supplementary Table S2: Confidence interval (CI) and power calculation (t-test or one-way ANOVA) for single cell experiments shown in Figure 2. Provided are sample size (N), significance level (α), effect size (d) or (f), and power.

Supplementary Table S3			CTL	DSS	DSS_R
Figure 3	3b	Lower 95% CI	39.7	24.0	31.2
		Upper 95% CI	44.1	29.1	35.3
		CTL vs. DSS: N=49, α = 0.05, f = 1.61, power > 95%			

Supplementary Table S3: Confidence interval (CI) and power calculation (one-way ANOVA) for single cell experiments shown in Figure 3. Provided are sample size (N), significance level (α), effect size (f), and power.

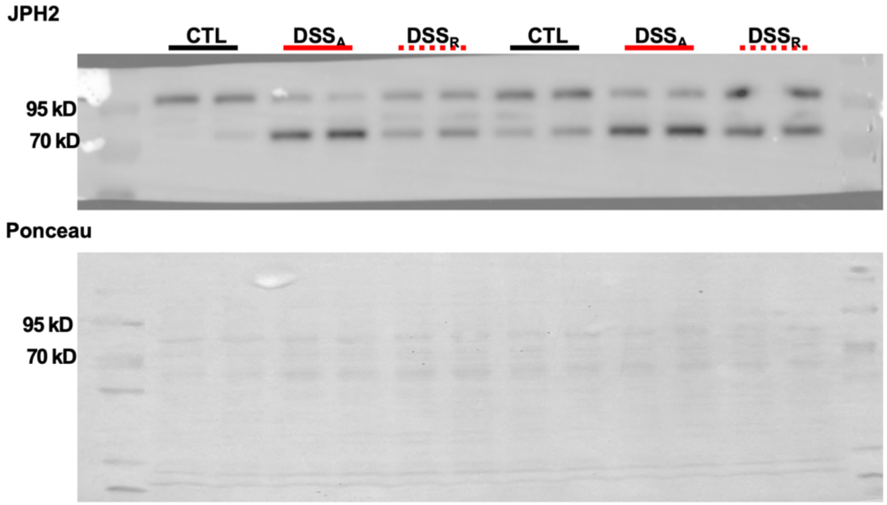

Supplementary Figure S1: Original, un-cropped blot shown in Figure 3c (top) and corresponding Ponceau staining of the same blot, used for normalization of protein expression.

Supplementary Table S4			CTL	DSS	$DSS_{I(ACE)}$
Figure 4	4d	Lower 95% CI	0.008	0.016	0.008
		Upper 95% CI	0.015	0.027	0.013
		CTL vs. DSS: N=26, α = 0.05, f = 0.83, power > 95%			

Supplementary Table S4: Confidence interval (CI) and power calculation (one-way ANOVA) for single cell experiments shown in Figure 4. Provided are sample size (N), significance level (α), effect size (f), and power.

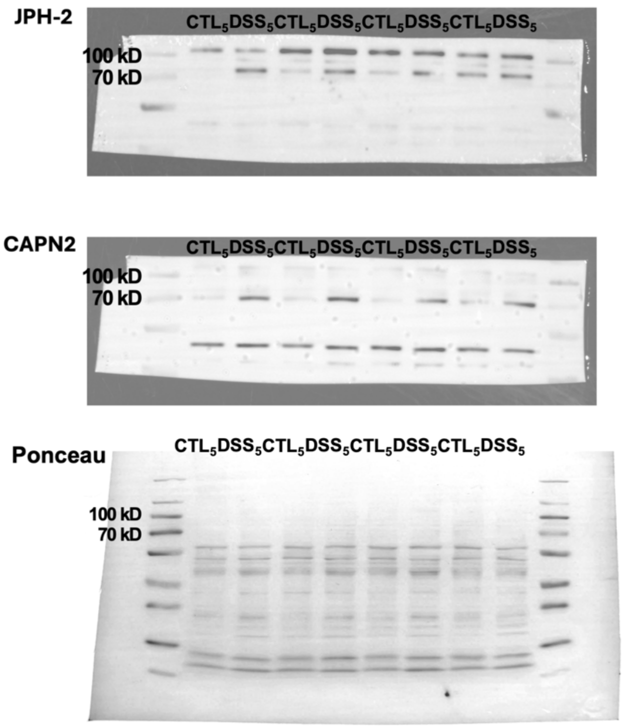

Supplementary Figure S2: Original, un-cropped blot shown in Figure 4e and corresponding Ponceau staining of the same blot, used for normalization of protein expression.

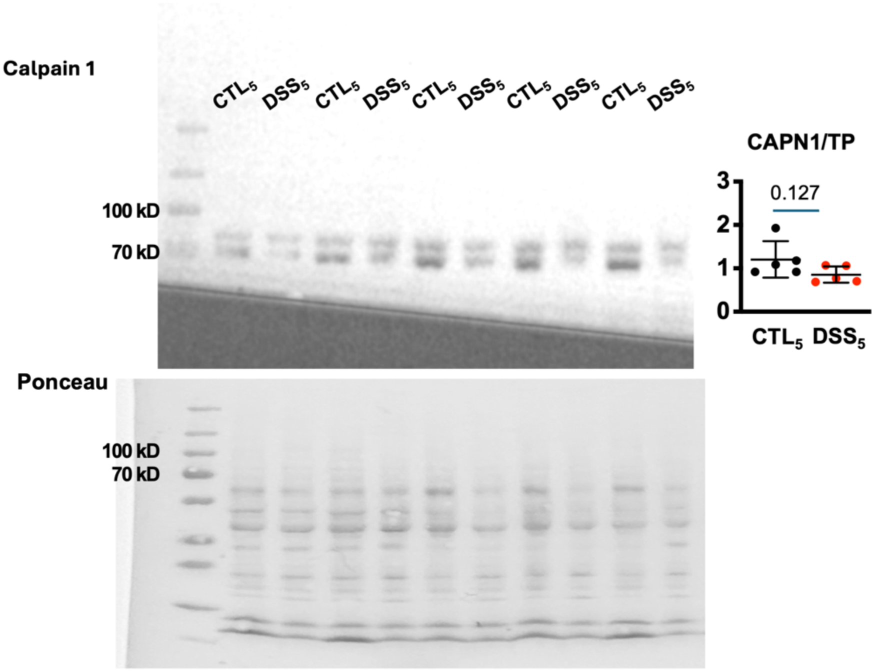

Supplementary Figure S3: Original, un-cropped blots showing CAPN1 protein levels in ventricular tissue from CTL and DSS animal (top) after 5 days of DSS treatment (DSS_5). Quantification of total CAPN1 is shown left, normalized to total protein (TP) obtained from Ponceau staining (bottom).

Supplementary Table S5			CTL	DSS	$DSS_{i(ACE)}$
Figure 5	5b	Lower 95% CI	24.0	41.2	38.3
		Upper 95% CI	29.1	45.9	40.9
		N=39, α = 0.05, f = 2.18, power > 95%			
	5c	Lower 95% CI	3.995	1.157	2.445
		Upper 95% CI	6.578	2.746	3.437
		N=50, α = 0.05, f =0.83, power > 95%			
	5d	Lower 95% CI	0.035	0.044	0.050
		Upper 95% CI	0.046	0.065	0.058
		DSS vs. $DSS_{i(ACE)}$: N=52, α = 0.05, d = 0.51, power > 85%			

Supplementary Table S5: Confidence interval (CI) and power calculation (one-way ANOVA) for single cell experiments shown in Figure 5. Provided are the sample size (N), significance level (α), effect size (f), and power.

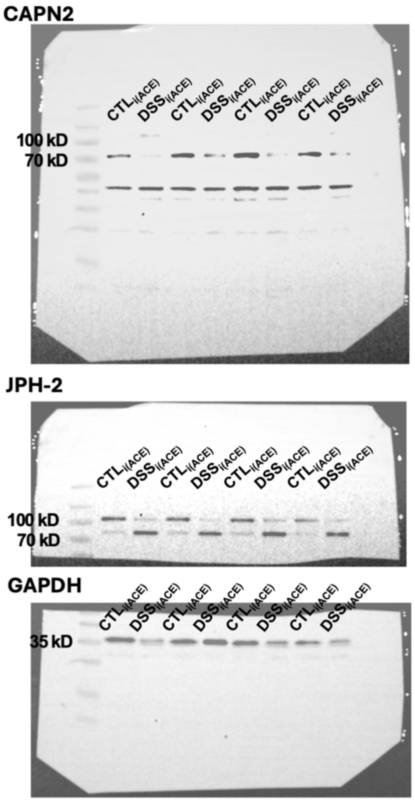

Supplementary Figure S4: Original, un-cropped blots (CAPN2, JPH-2) shown in Figure 5e and corresponding GAPDH staining of the same blot, used for normalization of protein expression.

Supplementary Table S6			CTL	CTL+Rapa	DSS	DSS+Rapa
Figure 6	6b	Lower 95% CI	16.4	67.2	71.4	46.0
		Upper 95% CI	34.2	98.5	96.5	93.3
		N=72, α = 0.05, f = .81, power > 95%				
			CTL	DSS	$DSS_{i(ACE)}$	
	6c	Lower 95% CI	16.38	71.36	31.20	
		Upper 95% CI	34.16	96.49	48.80	
		N=51, α = 0.05, f = 1.8, power > 95%				

Supplementary Table S6: Confidence interval (CI) and power calculation (one-way ANOVA) for single cell experiments shown in Figure 6bc. Provided are sample size (N), significance level (α), effect size (f), and power.

LC3B

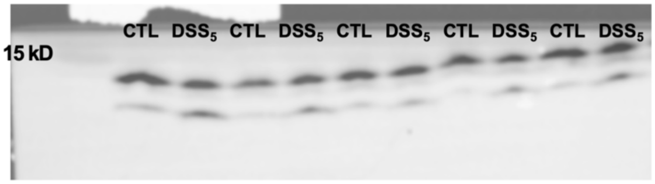

Ponceau

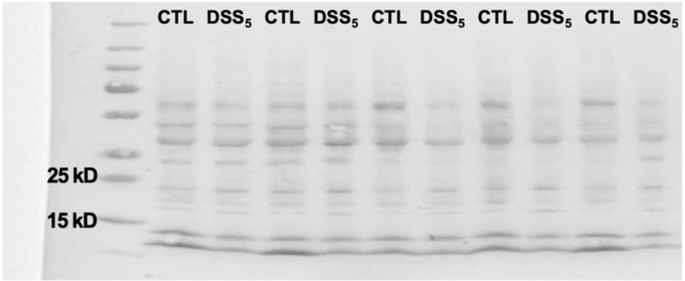

Supplementary Figure S5: Original, un-cropped blot (LC3B) shown in Figure 6d and corresponding Ponceau staining of the same blot, used for normalization of protein expression.
